# Supplementary figures and images for: Current knowledge of the Southern Hemisphere marine microbiome in eukaryotic hosts and the Strait of Magellan surface microbiome project
Source: PeerJ. 2023 Oct 3;11:e15978. doi: 10.7717/peerj.15978 (PMC10557944; doi:10.7717/peerj.15978)

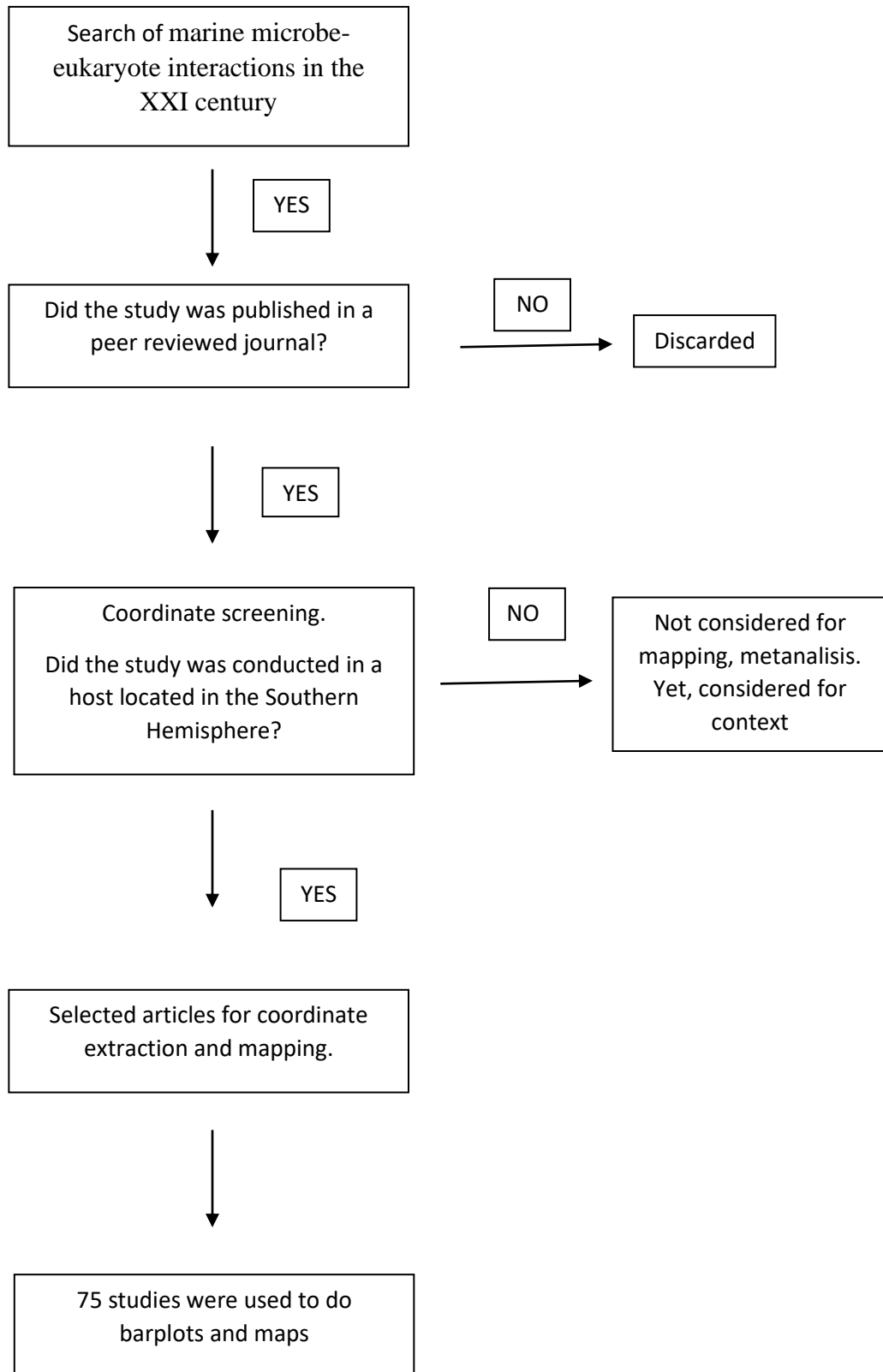

Supplement: Supplemental Information 1 [file peerj-11-15978-s001.pdf]
